# Notes and News

**Published:** 1892-03-05

**Authors:** 


### Notes and News.

Collected and Communicated.

At the annual meeting of the Great Northern Central Hospital attention was drawn to the want of accommodation, and the great need of a convalescent home was again alluded to. Were it not for the kindness of the authorities of several convalescent institutions in receiving patients from this hospital, the want would be more pressing than it is. Efforts are being made to correct the abuse of the institution in the out-patients' department, and a Casualty Officer has been appointed for the special supervision of this branch of the work. The Committee regret to announce an excess of expenditure over income exceeding that of the previous year. This is owing to a decrease in the amount of donations. The actual increase in general expenses during the past year has been but small. There still remains a sum of £14,000 for the building fund to be raised.

A letter appears in the *Globe* this week signed "M. D. Olim, in Oriente," which may possibly make some of the anti-opium army pause and ponder. The writer, who states he has been several years in charge of Government hospitals in the Straits Settlements, and has formed the decided opinion that opium smoking is beneficial rather than otherwise, climatic conditions being taken into account. He states that people of all nations require some tonic. Opium does some harm, not much, and it is rare to see a drunken Chinese coolie. He concludes his defence of opium by saying "it helps to keep off fevers, and to give tone to the system after a day's work in tropical countries, whilst in China itself its evils are not comparable to those caused by alcohol in England." If these facts do not admit of denial, a large number of people have been agitating themselves and others for a very doubtful reform.

Provincial hospitals needing a hand ambulance should communicate with Mr. Thomas Ryan, the Honorary Secretary of the Hospitals' Association London Street Ambulance Service, 140, Strand, W.C. The service has a number of Ashford Litters, in good condition, for which it has no longer use, and Mr. H. L. Bischoffsheim, the Treasurer, has kindly decided to present them to any Provincial hospitals needing such a conveyance.

The Royal Free Hospital, Gray's Inn Road, held its annual meeting on the 25th ult. A special court was also held at the close of the general meeting to consider the application that it is proposed to make for the grant of a Royal Charter of incorporation for the hospital. The President, Lord Dufferin, has written to express his approval of the proposal, and this letter was read to the court, wherein he states that the charter will doubtless be of the greatest value to the institution. The management and the public cannot fail to agree with him, and to further any scheme for the furtherance of so admirable an institution. The annual report, whilst showing the excellent work done, places before us an expenditure per bed less than that of any other general hospital in the Metropolis. Where efficiency and economy are combined the public may reasonably listen to appeals for much-needed support. Before the termination of the meeting, Lord Derby, Lord Latham, Lord Jaye, Lord Sele, and Sir Julian Goldsmid, M.P., were elected Vice-Presidents, whilst Mr. Gainsford Bruce, Q.C., M.P., was elected a Trustee of the hospital.

The proposed extension of the Royal Infirmary, Edinburgh, is provoking a good deal of discussion. The motives for the extension are mixed. Firstly, the patients need more room; and secondly, the students need more patients. A long article has been contributed to the *Scotsman*, which deals with the whole subject. The writer contends that Edinburgh has more free provision for its sick poor than either London or Manchester. He suggests that a distribution of students and teachers over the hospitals not now used for teaching purposes should be made. And, lastly, he considers that the enlargement of the Infirmary for the admission of lady students to departments reserved exclusively for their use should not be entertained, because, he says, circumstances indicate that male and female students will receive instruction side by side in the future. It seems to us that the argument that the difficulty of distributing students and the necessary increase of the teaching staff entails presents grave difficulties on the score of expense alone. It would be difficult to enforce attendance on the lectures of any particular man, and consequently some teachers would find themselves discoursing to overflowing classes, whilst others would be lecturing to empty benches. Then, again, as to the statement that men and women, or, more correctly speaking, boys and girls, are to be expected in the future to receive their education together. We venture to think that indications go very much the other way. Now that the claims of women to adequate technical training of many kinds is more generally admitted day by day, we see provision being made in many directions for their use alone, such as has long been the privilege of their brothers to enjoy. Hospitals for women, with female instructors and students, &c.; and now the mere suggestion that a part of the Royal Infirmary should be reserved for the instruction of lady students, and that the proposal should have been adopted with but little opposition, goes to show that the claims of women as separate factors are definitely admitted. We cannot regard the writer as a loyal citizen, should he desire to see a jewel in the crown of Edinburgh institutions less complete than it is possible to make it.

A LARGE meeting of Governors of the London Hospital was held on Wednesday. Mr. Buxton took the chair, and in moving the adoption of the report, referred to the attacks which had been made upon the nursing arrangements of the hospital. He said that it was impossible that the attacks could be continued; it was a question of a few people out of 3,000 insisting on small changes being made; the small points raised being more fitted for the House Committee. During the last year 9,458 in-patients had been treated, and so adaptable was the nursing system that not a single complaint had been made; everything had worked smoothly, and yet these are the arrangements which a few Governors wish altered. Yet the hospital has been working under a desire of some to find fault, which tends to encourage insubordination, and this is very unfair to the Committee of Management. It had come to be a case of "Who was to manage the London Hospital?" The adoption of the report was seconded by Mr. Martineau. Mr. Hutton gave his personal experience of the excellence of the private nurses. Mr. Hunter rose and said that no attacks had been made on the hospital; but that they had said, they did say, and they would continue to say, that the nurses in the hospital were overworked, and many changes were necessary. Mr. Kitto made a calm and dispassionate statement with regard to the nursing arrangements ending up by moving the following resolution: "That this meeting desires to express its unabated confidence in the administration of the hospital, its warm appreciation of the work of the House Committee, and its cordial thanks to the officers and nursing staff for their loyal co-operation." This was carried unanimously, and with loud and prolonged applause. Mr. Burdett rose to correct a few of Mr. Hunter's facts, stating that Mr. Hunter had not the technical knowledge necessary to enable him to form a correct judgment on the subject. There was a sense of proportion in all matters, and to reiterate petty charges was a reflection on the good feeling and common sense of those concerned. The report was adopted unanimously, Mr. and Mrs. Hunter and Mr. Yeatman walking out of the room just before the resolution was put, were greeted with derisive cheers by the Governors. After such a signal and complete defeat it is hoped that the malcontents will recognise their error, and combine with the majority of the Governors at future quarterly courts to promote and not to impede the well-being of the hospital. There was great rejoicing throughout the entire hospital, especially amongst the nursing staff, at the result of the court.

